# Global Emergence of Infectious Bronchitis Virus Variants: Evolution, Immunity, and Vaccination Challenges

**DOI:** 10.1155/2023/1144924

**Published:** 2023-11-29

**Authors:** Hassanein H. Abozeid

**Affiliations:** ^1^Department of Poultry Diseases, Faculty of Veterinary Medicine, Cairo University, Giza 12211, Egypt; ^2^Department of Pathobiological Sciences, School of Veterinary Medicine, University of Wisconsin-Madison, Madison, WI 53706, USA

## Abstract

Infectious bronchitis is an acute, extremely contagious viral disease affecting chickens of all ages, leading to devastating economic losses in the poultry industry worldwide. Affected chickens show respiratory distress and/or nephritis, in addition to decrease of egg production and quality in layers. The avian coronavirus, infectious bronchitis virus (IBV), is a rapidly evolving virus due to the high frequency of mutations and recombination events that are common in coronaviruses. This leads to the continual emergence of novel genotypes that show variable or poor crossprotection. The immune response against IBV is complex. Passive, innate and adaptive humoral and cellular immunity play distinct roles in protection against IBV. Despite intensive vaccination using the currently available live-attenuated and inactivated IBV vaccines, IBV continues to circulate, evolve, and trigger outbreaks worldwide, indicating the urgent need to update the current vaccines to control the emerging variants. Different approaches for preparation of IBV vaccines, including DNA, subunit, peptides, virus-like particles, vectored and recombinant vaccines, have been tested in many studies to combat the disease. This review focuses on several key aspects related to IBV, including its clinical significance, the functional structure of the virus, the factors that contribute to its evolution and diversity, the types of immune responses against IBV, and the characteristics of both current and emerging IBV vaccines. The goal is to provide a comprehensive understanding of IBV and explore the emergence of variants, their dissemination around the world, and the challenges to define the efficient vaccination strategies.

## 1. Infectious Bronchitis

Infectious bronchitis (IB) is an acute viral disease of chickens that causes devastating economic losses in the poultry industry worldwide [1]. All ages of chickens are susceptible to infection. IB has a short incubation period ranging from 16 to 48 hr. It is extremely contagious and can spread very rapidly from the infected chickens to the entire flock, both directly by aerosol and indirectly by mechanical means [2].

**Figure 1:**
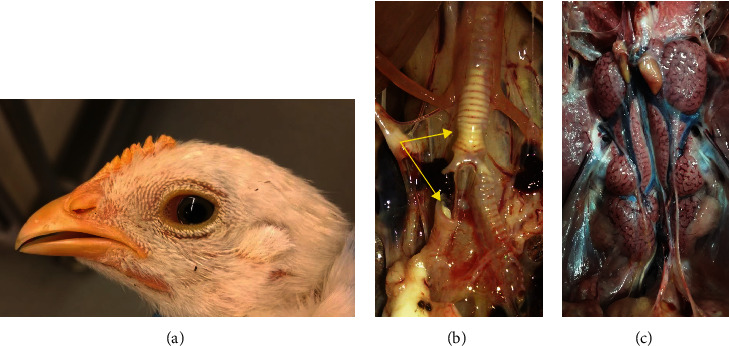
Clinicopathology of infectious bronchitis in broilers. (a) Lachrymation; (b) caseous exudate forming a plug at the tracheal bifurcation; and (c) pale, swollen kidney with renal tubules filled with ureates.

Depending on the organ/system affected and the tropism of the infecting strain, IB is manifested clinically in three major forms, namely, respiratory, renal, and reproductive. Respiratory signs are characterized by nasal discharge, lacrimation (Figure 1(a)), sneezing, cough, tracheal rales, and gasping [2]. Mortality is usually low but can reach 20%–30% when secondary bacterial complications by *Escherichia coli* and mycoplasma occur [3]. On necropsy, congested trachea with serous, catarrhal, or caseous exudate could be found. Accumulated caseous exudate could form a caseous plug at the tracheal bifurcation leading to asphyxia, which is the main cause of death (Figure 1(b)). When a secondary bacterial infection occurs, caseous pericarditis, perihepatitis, and air saculitis are usually observed [3].

Renal signs caused by nephropathogenic IBV strains, such as 4/91, B1648, Aus T, QX-like, and TW are characterized by depression, ruffled feathers, excessive water intake, and wet, whitish droppings with a high amount of ureates [4–13]. In infected young chickens, mortality can reach 20%–25% [12, 13] and increases up to 1% weekly in case of urolithiasis [3]. Some managemental factors such as cold stress, high dietary protein or calcium, and elevated water hardness can exacerbate the nephropathogenic effect of IBV [4, 14]. Older chickens are more resistant to the nephropathogenic effect of IBV [9, 15–17]. On necropsy, pale, marbled, and swollen kidneys with the tubules and ureters distended with ureates are commonly observed (Figure 1(c)) [6, 12, 13, 18, 19].

**Figure 2:**
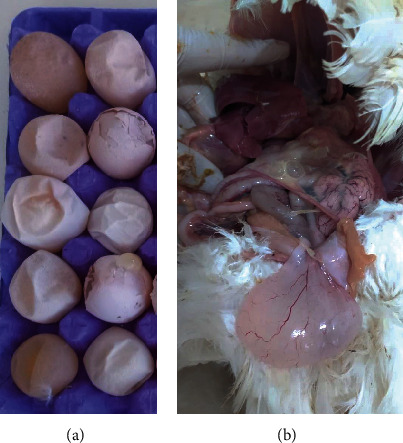
Clinicopathology of infectious bronchitis in layers. (a) Deformed eggs including soft shell, thin shell, and shell-less eggs; (b) cystic oviduct.

In layers, marked decline in egg production is observed [20, 21]. The produced eggs are of inferior quality; pale, soft-shelled, rough-shelled, shell-less, small sized, and/or misshapen (Figure 2(a)) [22]. The internal quality of the produced eggs is also affected, with eggs exhibiting watery albumin with no clear demarcation between the thick and thin albumin [22]. The extent of production decline can vary from mild to as much as 70% based on the stage of lay at infection, the infecting strain, and the immune status [2, 23]. QX-type IBV was shown to induce more pathogenic changes in the oviduct in laying stage compared to the Mass-type IBV [24]. When young chicks are infected with IBV, permanent damage of the oviduct occurs resulting in failure of production when the chickens come into maturity (false layers) [25, 26]. On necropsy, chickens consequently show cystic oviduct (Figure 2(b)) [10, 21, 22, 25–28]. Egg peritonitis is also seen due to the shortness and narrowing of the oviduct leading to internal laying [3, 22].

## 2. Infectious Bronchitis Virus

### 2.1. Viral Genome and Proteins

**Figure 3:**
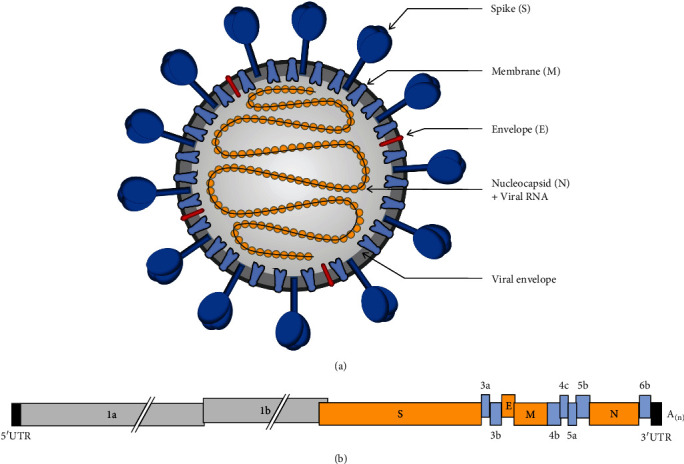
Schematic diagram of infectious bronchitis virus. (a) Virus structure and (b) virus genome. Replicase gene composed of ORFs 1a and 1b is shown in gray; genes encoding for structural proteins S, E, M, and N are shown in orange; genes encoding for the accessory proteins 3a, 3b, 4b, 4c, 5a, 5b, and 6b are shown in blue. 5′ and 3′ untranslated regions (UTR) are shown in black; *A*(*n*) denotes the poly-A tail.

Infectious bronchitis virus (IBV) belongs to group 3 of the *coronavirus* genus in the *Coronaviridae* family. IBV is an enveloped virus of about 120 nm in diameter and possesses large club-shaped spikes of about 20 nm in length [29] (Figure 3(a)). The genome of IBV is a single-stranded, positive-sense RNA of approximately 27.6 Kb comprising as many as 13 open reading frames (ORFs) in the order 5′-UTR-1a-1b-S-3a-3b-E-M-4b-4c-5a-5b-N-6b-UTR-Poly(A)tail-3′ (Figure 3(b)). Gene 1, the replicase gene, extends for about two-thirds of the viral genome and is composed of ORFs 1a and 1b [30]. Gene 1 encodes for polyproteins pp1a and pp1ab that are autoproteolytically processed into 15 nonstructural proteins (nsp2–nsp16) involved in viral genome replication and transcription. Some of these nonstructural proteins have the ability to suppress both innate and adapted immune responses (reviewed in Peng et al. [31]). The replicase gene is also shown to be a determinant of viral virulence [32–34].

The remaining one-third of the viral genome encodes four structural proteins: spike (S), envelope (E), membrane (M), and nucleocapsid (N), interspersed by as many as seven accessory proteins: 3a, 3b, 4b, 4c, 5a, 5b, and 6b (Figure 3(b)). These accessory proteins are not essential for viral replication [35, 36]. However, they have been suggested to play a role in antagonizing the host innate immunity and contribute to viral pathogenicity [31, 37, 38]. It has been shown that deletion of the accessory genes 3a, 3b, 5a, and/or 5b in IBV resulted in mutant viruses with attenuated characteristics [39, 40]. Zhao et al. [41] further demonstrated a greater effect of protein 3b on pathogenicity than protein 3a. In addition, the virulent YN strain became attenuated after replacement of the 5a accessory protein with that of the attenuated aYN strain [42]. The protein 5a also plays an important role in virus–host interaction [43].

The S protein is a highly glycosylated type I transmembrane glycoprotein presented as a trimer on the surface of the virion particles. The molecular mass of the S protein monomer before glycosylation is about 128 kDa, which increases to about 200 kDa after it obtains extensive *N*-linked glycosylation in the endoplasmic reticulum (ER) [44]. The S glycoprotein is the major immunogenic protein eliciting the protective immune responses. The S protein is cleaved post-translationally into S1 and S2 subunits. The S1 subunit (∼520 aa) is the aminoterminal part forming the globular head, while the S2 subunit (∼625 aa) is the carboxyl-terminal part forming the stalk domain anchored in the viral membrane [45]. The S1 subunit is the most variable part and is responsible for the viral attachment, tissue tropism, and induction of serotype-specific antibodies as it harbors the receptor binding domains (RBD) and the major serotype-specific neutralizing epitopes. The greatest diversity of the S1 nucleotide sequence is mostly found within three key hypervariable regions (HVRs): HVR1 (amino acid residues 38–67) and HVR2 (amino acid residues 91–141) in the *N*-terminal domain (NTD), and HVR3 (amino acid residues 274–387) in the C-terminal domain (CTD) [46, 47]. Both NTD and CTD of the S1 subunit have been proposed to be associated with receptor binding [48]. The S1-NTD binds to *α*-2,3 linked sialic acid receptors on chicken respiratory epithelia [49]. Bouwman et al. [50] showed that amino acids in HVR2 of QX-RBD are critical for receptor binding. The S1-CTD harbors two extended loops in its core structure, which function as receptor-binding motifs (RBMs) and have been shown to interact with an unidentified receptor [48]. The spike neutralizing epitopes are highly conformation-dependent that are formed by the interplay between S1 and S2 subunits [51]. The S2 subunit is a highly conserved part formed primarily by the heptad repeat regions, HRP1 and HRP2, and a fusion peptide that is responsible mainly for the viral entry and membrane fusion [48]. Research has shown that the S2 subunit is responsible for viral adaptation to various cell lines [52–58]. The S2 subunit also contains some minor neutralizing epitopes and contributes to the avidity and specificity of virus attachment, and thus viral host range [51]. In addition, the S protein has been shown to play a role in determining the pathogenicity of IBV [42].

The M protein is the most abundant viral structural protein embedded in the viral envelope and is usually observed as a dimer. The molecular mass of the M monomer is 25–30 kDa [29]. The E protein, also named small envelop protein, is the less abundant protein in the viral envelope of 8–12 kDa [59]. The E protein is critical for viral infectivity. The M and E proteins are essential for the assembly and viral budding from the ER [29]. The M and E proteins have been shown to be sufficient for the production of virus-like particles [60].

The N protein is an internal phosphoprotein that presents as monomers (45–50 kDa) that bind to the viral genomic RNA, forming the helical nucleocapsid structure. The N protein plays a crucial role in viral replication and assembly [29] and has been reported to play a key role in cellular immunity due to the presence of cytotoxic T lymphocyte (CTL)-inducing epitopes located at its carboxylic terminus [61–63]. B-cell epitopes are also reported in N protein [64].

### 2.2. Classification of IBV Strains

Classification of IBV strains is useful for implementation of control strategies for IB. Knowledge of specific serotypes/genotypes circulating in each geographical region can enable determination of the epidemiology and evolution of IBV strains circulating in the field.

**Figure 4:**
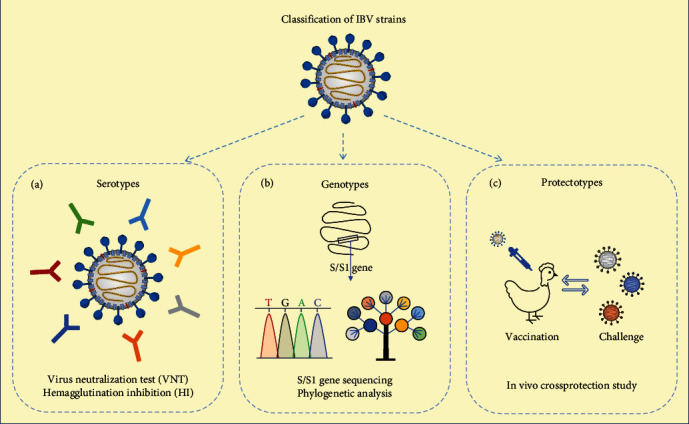
Classification of infectious bronchitis virus (IBV) strains into: (a) serotypes, using virus neutralization test and/or hemagglutination inhibition assays; (b) genotypes, using S or S1 gene sequencing and phylogenetic analysis; and (c) protectotypes, using in vivo crossprotection studies.

Genotyping of IBV strains has been established based on the nucleotide sequences of the S1 gene using reverse transcriptase-polymerase chain reaction (RT-PCR) followed by DNA sequencing [65] (Figure 4(b)) or restriction enzyme fragment length polymorphism [66]. RT-PCR using genotype-specific primers can be used for detection and differentiation of IBV genotypes [67–73]. To standardize IBV genotype classification, Valastro et al. [74] strongly recommended using the whole S1 gene for phylogenetic analysis. Interestingly, phylogenetic analysis using partial S1 gene sequence, including HVRs 1 and 2 and HVR3, may produce inconsistent clusters compared to full S1 gene sequence, owning the risk of misclassification.

IBV strains have been classified into serotypes based on the antigenicity of the S protein using virus neutralization (VN) or hemagglutination inhibition (HI) assays (Figure 4(a)). Two IBV strains are considered of the same serotype when the two-way heterologous neutralization titers differ less than 20-fold from the homologous titers in both directions [75].

Classification of IBV strains into protectotypes or immunotypes is primarily based on in vivo crossprotection studies (Figure 4(c)). This classification system gives reliable information about the efficacy of IBV vaccines. IBV strains that provide protection against each other are of the same protectotype. Such crossprotection is presumably due to some common epitopes shared between different genotypes or serotypes [76, 77]. The immunological aspects related to protectotypes are further discussed in the immunity of IBV section.

Unfortunately, the correlation between genotypes (S1 amino acid identity) and serotypes (VN), and protectotypes (in vivo crossprotection) is not consistent. Generally, IBV strains belonging to the same serotype show more than 95% amino acid identity in the S1 subunit [78], while IBV strains of different serotypes show less than 85% amino acid identity in the S1 subunit [67, 79]. However, some IBV strains, defined to be of the same serotype by VN, commonly differed by about 20%–25% amino acid identity and sometimes, by up to 48% amino acid identity. Moreover, strains showing 97% amino acid identity were classified by VN to be of different serotypes [80]. That is because as few as 2%–3% difference of amino acids within the three HVRs in the S1 subunit, specifically those involved in formation of the neutralizing epitopes, can change the serotype and hence the level of crossprotection [80, 81]. This explains why some IBV strains with a very high homology of S1 protein (96%–98%) provided limited crossprotection [76], whereas some other IBV strains with a lower homology may show a higher level of crossprotection [82, 83]. For example, Massachusetts and Connecticut strains of IBV are clustered within the same genotype (GI-1), but they were identified as different serotypes (antigenically different), that is, they do not crossneutralize [84]. Similarly, Massachusetts and Beaudette strains of IBV are clustered within the same genotype (GI-1) with more than 96% aa identity in their S1 genes. However, antibodies raised against the Beaudette strain were unable to neutralize the Massachusetts strain in vitro using VN, or provide protection in challenged chickens [85]. Conversely, a Japanese JP-III vaccine sharing 83.1% amino acid identity with the Japanese QX-like JP/ZK-B7/2020 strain showed more than 2.0 VN titer and provided high protection against challenge in SPF chickens [86]. Therefore, serotyping, and genotyping should be carried out together for accurate classification outcomes [87].

### 2.3. Diversity and Evolution

Several serotypes and genotypes of IBV have been reported worldwide [1, 84]. The diversity of IBV serotypes is mainly attributed to the variability of the S protein. The S1 subunit has shown amino acid differences ranging from 20% to 50% between IBV strains [83, 88]. Like most RNA viruses, mutations and genetic recombination are the major events responsible for the diversity and evolution of avian coronavirus IBV. Mutations are generated during viral replication due to the lack of a proofreading mechanism of the viral RNA polymerase [89]. Being a fast-replicating virus with a short generation time and a large RNA genome, IBV is prone to acquiring mutations and accommodating genetic recombination. Because of the relevance of the S protein for the virus immunological escape, genetic drift directed toward the S protein is suggested to be the most relevant feature driving the viral adaptive evolution for survival. Genetic recombination has been reported in coronaviruses including IBV [90–92]. The unique discontinuous transcription mechanism of coronaviruses favors the generation of recombinants resulting from the random template switching mechanism of the viral polymerase [93–95]. When two different IBV strains infect the same cell, the generation of recombinant viruses is very likely to occur, leading to the emergence of new variants [96]. These new variants are mostly distinct from the parental strains and can often escape the pre-existing immunity, inducing outbreaks in vaccinated flocks [97–99].

Some regions in the viral genome, called recombination hot spots, have been reported for a higher incidence of recombination events. These regions encode for nsp2, nsp3, nsp16, and the S glycoprotein [100]. Because the nonstructural proteins encoded by the ORF 1ab are associated with viral pathogenicity, the emergent IBV strains with recombinant nonstructural proteins could be of altered pathogenicity and increased virulence. On the contrary, genetic recombination could also lead to the emergence of low virulence variants [90, 101]. As the S protein is a determinant of viral tropism and contains the major neutralizing epitopes, recombination in the S protein could lead to the generation of not only a new serotype, but also a new virus of altered tropism and host specificity. This was documented for the turkey coronavirus (TCoV) that was suggested to have emerged by replacement of the S protein in IBV with a yet unknown sequence, probably from another avian coronavirus [102]. This study revealed that both IBV and TCoV showed more than 86% full-genome nucleotide similarity while the S protein of both viruses showed less than 36% nucleotide similarity, with potential recombination breakpoints at the upstream and the 3′ end of the S gene. Recently, recombination events between IBV and TCoV have also been recorded [103, 104]. In addition, recombination of S genes from different genotypes also could result in the emergence of new genotypes/lineages [105].

Due to the high mutation rate, coronaviruses including IBV usually exist as a mixture of subpopulations within an isolate or a vaccine [106–111]. It has been reported that the invading vaccine population is subjected to positive selective pressure according to the microenvironment of distinctive host tissues to select the fittest population [112]. Selective pressure could also be exerted using live-attenuated IBV vaccines that provide partial protection and thus allow some viruses to replicate and persist in the vaccinated flocks. This could result in further adaptation and evolution to escape immunity [113–116], leading to a faster evolutionary rate [117, 118]. For example, the IBV GA98 was suggested to have emerged under the immune selection pressure exerted by the use of the DE072 vaccine, and showed fast evolutionary and mutation rates of 2.5% and 1.5% per year, respectively, in the HVR [116] compared to 0.3%–0.6% mutation rate per year for the 793/B genotype in the absence of vaccine use [119]. Under experimental conditions, Flageul et al. [120] demonstrated that IBV D388 evolved rapidly over three passages in both unvaccinated chickens and chickens vaccinated with the heterologous vaccine strain H120. However, the viral population selection and genetic drift were completely different in both groups.

## 3. Emergence and Dissemination of IBV Variants Worldwide

In 2016, Valastro et al. [74] classified IBV strains into six main genotypes (GI through GVI) based on the full S1 gene sequence. These genotypes encompass 32 lineages, with 27 lineages identified within genotype GI and one lineage each within the remaining genotypes. In addition, 26 unique variants (UVs) were identified that did not cluster within any of the established lineages. Subsequently, as more IBV S1 gene sequences became available, some of the UVs were identified as new genotypes. Moreover, novel IBV variants emerged in different parts of the world and have been assigned new genotype classifications as well. To date, at least nine well-established genotypes (GI through GI-IX) have been identified encompassing 41 lineages (31 lineages within GI, 2 lineages within each of GII and GVIII, and 1 lineage within the remaining genotypes). Confusingly, some of the reported UVs were designated tentatively as novel genotypes based on only one or two S1 gene sequences. However, according to the harmonized classification system proposed by Valastro et al. [74], the S1 gene sequence of at least three viruses collected from two different outbreaks are required to establish a new genotype/lineage. This section explores the updated global landscape of IBVs, the wide dissemination of IBV variants, and the emergence of novel genotypes/lineages.

### 3.1. The Global Landscape of IBV Variants

In the 1930s, IBV was first recorded in diseased chicken flocks in the USA [121]. This initial strain, known as the classic Mass-type, was later classified as part of the GI-1 lineage, including H120, H52, Connecticut, Beaudette, and classic-like field isolates. Over time, IBV strains belonging to the GI-1 lineage have spread extensively across the globe, likely due to their widespread use as vaccines in poultry production. 793B-types (later identified as GI-13) has also spread to several countries, due to being used as vaccines along with the Mass-type in heterologous vaccination programs, known as “protectotype.” Since IBV is a rapidly evolving virus, several variant strains have emerged over the years in face of the intensive vaccinations.

**Figure 5:**
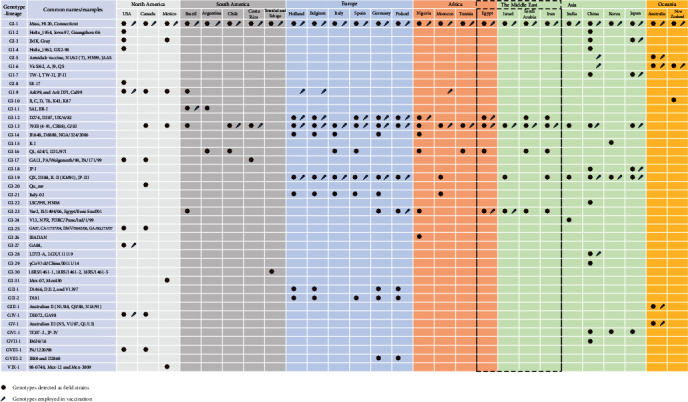
Global landscape of IBV genotypes and lineages. A virus icon indicates the presence of field viruses. The vaccine icon indicates the genotypes employed in vaccination in each respective country. Dashed borders delineate the Middle East region.

While certain variants have exhibited global prevalence, others have been identified as indigenous to specific geographical regions, suggesting a localized transmission and adaptation (Figure 5). For example, in North America, various indigenous genotypes have been identified, including GI-8, GI-9, GI-17, GI-20, GI-25, GI-27, GIV-1, GVIII-1, with GI-20 reported exclusively in Canada [74]. Interestingly, GI-17 has been isolated recently from outbreaks during 2016–2017 in Costa Rica [122]. Mexico is endemic with GI-31 and GIX [123] (discussed later in this review). South America is characterized by the presence of the indigenous GI-11 lineage [74] in addition to GI-30 recently identified in Trinidad and Tobago [124]. In Asia, several indigenous lineages have been reported including GI-7, GI-15, GI-18, GI-22, GI-24, and GVI-1, with GI-15, GI-22, and GI-24 exclusively reported in Korea, China, and India, respectively [74]. Recently, GI-28, GI-29, and GVII-I have been identified in China [125, 126]. Australia and New Zealand are home to the indigenous lineages GI-5, GI-6, GI-10, GIII-1, and GV-1 [74, 127]. The African continent harbors the indigenous GI-26 lineage, observed in Nigeria and Niger [74, 128]. Europe has been identified to host the GI-21, GII-1, GII-2, and GVIII-2 lineages [74, 129]. GI-21 has been reported in Morocco as well [130]. GI-2, GI-3, and GI-4 have been reported to be confined primarily to North America and Asia [74]. While some of these indigenous genotypes/lineages were observed for a short period of time, others became predominant within their respective regions. For instance, GI-2, GI-4, and GI-8 were detected only in a limited period in the USA, indicating its limited importance [74]. On the other hand, GI-9 (ArkDPI), GI-17 (DMV/1639), GI-27 (GA08), and GIV-1 (DE072) became predominant in the USA [131]. In China, detection of GI-2, GI-3, and GI-4 became rare while detection of GI-7, GI-22, and GI-28 (LDT3-like) is increasing [132]. GI-23 was considered indigenous to the Middle East for approximately two decades since its emergence in the mid-1990s [74]. However, GI-23 has been recorded in several countries in Europe since 2016 [129, 133] and more recently in South America [134]. In addition, GI-16 and GI-19 are expanding their geographical distribution and spreading to multiple regions including Asia, Europe, Africa, and South America.

### 3.2. IBV Variants Expanding their Geographical Distribution

It is noteworthy that GI-16, GI-19, and GI-23 are disseminating to numerous countries, thereby expanding their geographical distribution. Although the origin, evolution, and spread dynamics are unique for each genotype, there are common causes that explain the methods of their widespread across several countries such as the live poultry trade, economic, and political relationships among countries and migratory birds. The intensive poultry industry, and the absence of serotype-specific immunity facilitated the spread of the introduced genotype within the country. Nonetheless, introduction of exogenous genotypes could also occur with the introduction of vaccine strains. In China, for instance, GI-5 and GI-6 were recorded after the introduction of JAAS and J9 vaccines from Australia [135]. Moreover, the detection of GI-13 field strains was recorded in Egypt [136], Chile [137], and Costa Rica [138] several years after the introduction of 793B live vaccines.

GI-16 (Q1-like), also known as 624/I, T3, J2, LDL/97I, Korean-III -like viruses, have been reported in several countries in Asia [139], Europe [129], South America [105, 140–143], Africa [144, 145], and the Middle East [146–148]. Although 624/I was initially identified in Italy in 1993, retrospective studies backdate its emergence to 1963 [149]. After gradual diminishing, the Q1 strain was reported in China in 1996 causing proventriculitis [150]. Retrospective studies revealed that both the Italian 624/I and the Chinese Q1 strains have the same origin nearly at the beginning or middle of the 20th century [151, 152]. A recent phylodynamic study revealed that GI-16 migrated from Italy to Asia, serving as the primary core for subsequent dissemination to the Middle East, Europe, and notably South America, probably facilitated by multiple introduction events [152]. The introduction of GI-16 from Europe to China might be attributed to the live breeders' trade, aimed to enhance the local genetic lines in China. The study also suggested backflow from China to Italy based on the detection of two Chinese Q1 strains in Italy. The migration of wild birds could also have played a role in the intercontinental virus transmission, especially over the short distance between Europe and Asia. However, the extreme long distance to South America suggests that other hypotheses such as undeclared animal trade were more likely to have established the introduction of GI-16 [152]. While the clinical impact of the Italian Q1 strains has been minimal in recent years, the introduction of the South American Q1 strain had a significant clinical and economic impact in those countries. This may be attributed to the genetic differences between both strains and/or different vaccination regimens implemented in both countries [141, 142, 152].

It is noteworthy that the introduction of the GI-16 lineage to South America resulted in extensive recombination with the indigenous GI-11 strains circulating in the region since the 1950s [105, 143]. This led to the emergence of novel recombinant GI-11 strains, mainly found in Argentina and Uruguay, which combine the ORF S of the native GI-11 with the backbone genome from the introduced European/Asian GI-16 lineage [105]. Currently, GI-16 is more prevalent in Western countries along the Pacific Ocean in South America [105, 142].

Introduction of GI-16 viruses from China to Egypt was predicted about in 2010 [152]. Currently, GI-16 has been also detected in other African countries such as Nigeria [144] and Ivory Coast [145]. Yet, little information is available about this lineage in Africa.

GI-19 (QX-type), also known as LX4, Korean-II, and Japanese-III-like, Dutch D388 viruses was first detected in China in 1996 [153]. Since then, GI-19 has been undergoing extensive evolution primarily through recombination [154, 155]. While many of these variants eventually disappeared due to their inability to thrive in chicken hosts, and other viruses that exhibited adaptability (“fitness”) continued to evolve and gradually gained prevalence in commercial poultry across China after 2006 [156]. Currently, GI-19 has been frequently reported in several countries in Asia, Europe, and Africa [6, 12, 20, 129, 153, 157–159]. A phylodynamic reconstruction of GI-19, utilizing 807 partial S1 sequences of strains collected from 18 countries, revealed that the GI-19 genotype originated in China more than 10 years before its initial identification in 1996, probably because of limited surveillance and/or reporting [158]. Following this extended period of local circulation, it potentially gained virulence and eventually emerged as a pathogenic genotype. The study revealed that GI-19 gradually spread to various countries plausibly in at least four successive waves: (1) introduction in Europe in the early 2000s, (2) spread in central Europe and Italy around 2005, (3) expansion in Spain and Eastern European countries approximately during 2008–2010, and (4) dissemination to the Middle East and North Africa during 2013–2015. These multiple waves of viral dissemination could have resulted in fluctuations in viral population size. The closer geographic and economic ties among European nations plausibly explain the fast spread of GI-19 among European countries. However, the colonization of new regions by the GI-19 lineage was primarily driven by single introduction events followed by local expansion [158]. Several well-established epidemiological links among distantly related regions indicate that animal transportation and indirect transmission routes, rather than local airborne diffusion, play a significant role in the local persistence of QX [158].

GI-23, also known as Variant-2, is expanding its geographical distribution after being considered an indigenous Middle Eastern strain for more than 10 years. GI-23 is now frequently reported in several countries in Europe such as Poland, Germany, Romania, Lithuania [129, 133], and more recently in Brazil in South America [134]. In 2021, Ekiri et al. [144] reported the detection of GI-23 in three broiler flocks and eight layer flocks in Nigeria using a Variant02-specific real-time RT-PCR kit. However, no S1 gene sequences are available to help determine the source of virus introduction.

A phylodynamic analysis revealed that GI-23 after originating in the Middle East in the 1990s, circulated undetected or underdiagnosed, possibly due to low virulence, limited poultry industry development, and/or poor diagnostic methods [160]. However, with the growth of the poultry industry, there was a gradual increase in viral population size between the late 1990s and 2010, leading to an increase in viral virulence. The study revealed a notable recombinant clade, exhibiting potential higher virulence and fitness, which appeared to spread from the Middle East to European countries. Turkey, functioning as an intercontinental “bridge” between less related countries, played a crucial role in facilitating this transmission [160]. The initial introduction of GI-23 to Europe was documented in Poland in late 2015 from a diseased broiler flock [161], followed by spread among multiple European countries including Germany, Lithuania, Romania, and Ukraine [129, 133].

In 2022, Ikuta et al. [134] reported the first introduction of GI-23 to South America in Brazil with distinct amino acid mutations compared to the GI-23 sequences from other continents. Phylogenetic analysis of 120 Brazilian GI-23 strains revealed the presence of two distinct subclades, namely SA.1 and SA.2. Intriguingly, both subclades clustered with the Eastern European GI-23 strains, suggesting possible introductions from Europe, likely through multiple events between 2017 and 2019 [162]. The expanding poultry market in Brazil, coupled with international trade, may have facilitated the introduction of the GI-23 genotype through the importation of live birds, fomites, and/or human movement [162].

### 3.3. Emergence of Newly Identified IBV Genotypes/Lineages

According to the standardized classification of IBV genotypes established in 2016, a novel genotype lineage should form a monophyletic group consisting of a minimum of three viruses collected from at least two different outbreaks, defined by strongly supported nodes (>0.98 SH-like test support value), and demonstrate uncorrected pairwise distance of not less than 13% in their S1 gene sequences [74]. Since 2016, newly identified genotypes and lineages have been reported in different parts of the world including GI-28, GI-29, and GVIII in China, GI-30 in Trinidad and Tobago, GI-31 and GIX in Mexico, and GII-2 and GVII-2 in Europe. Some UVs (less than three viruses) were also reported in Mexico and Ivory Coast.

GI-28: In 2017, Chen et al. [125] reported a distinct IBV strain (LGX/111119), designating it as a novel lineage within GI (GI-28). The reported IBV strain clustered closely with six reference strains previously classified as UVs, namely, the Chinese Variant-2 group [163], exhibiting 96% amino acid identity, while being distinct from all other identified genotypes/lineages. LGX/111119 strain was suggested to be emerged either as a result of recombination including the LX4 strain (GI-19) and an unknown IBV that contributed to the S1 gene, or by accumulation of mutations and selections in the S1 gene of the LX4 strain [125]. The Chinese strain LGX/111119 was also identified as a novel serotype after testing against five IBV strains antisera; Massachusetts, 793/B, LX4, ck/CH/LDL/97I, and TW-I serotypes. LGX/111119 induced nephropathogenic effects in 1-day-old SPF chicks and exhibited multiorgan tropism (respiratory, renal, digestive, and reproductive) [125]. Despite the introduction of the LDT3-A (GI-28) vaccine alongside the commonly used H120 and 4/91 vaccines since 2011, GI-28 remains prevalent in various geographic regions within China [164], indicating its endemic nature. Later, a new recombinant IBV strain CK/CH/MY/2020 belonging to GI-28 emerged because of multiple recombination events involving three live-attenuated vaccine strains: H120, 4/91, and LDT3-A [165]. Despite being composed of three attenuated vaccine strains, the recombinant IBV strain CK/CH/MY/2020 caused clinical disease, resulting in 40% mortality in SPF chickens. This finding highlights the risk associated with the use of multiple live-attenuated vaccines, as they may revert to virulence by recombination, even in the absence of the field strains. Moreover, recombination among the three vaccine strains and the field strain LJL/08-1 (GI-19) was also recorded [166].

GI-29: Jiang et al. [126] identified three Chinese IBV strains, namely *γ*CoV/ck/China/I0111/14, *γ*CoV/ck/China/I0114/14, and *γ*CoV/ck/China/I0118/14, which were isolated in 2014 from various outbreaks. These strains were found to be genetically and antigenically distinct viruses, leading to their classification as a novel lineage within GI (GI-29). GI-29 was suggested to have emerged from a GX-YL5-like virus through the accumulation of genome-wide substitutions. In addition, *γ*CoV/ck/China/I0114/14 exhibited evidence of two recombination events involving the vaccine strain 4/91, specifically within the nsp2 and nsp3 regions [126]. GI-29 also demonstrated nephropathogenicity, however, it did not induce cystic oviduct [126].

GI-30: In Trinidad and Tobago, Jordan et al. [124], molecularly characterized the three IBV strains 18RS/1461-1, 18RS/1461-2, and 18RS/1461-5 that were previously isolated in 2014 from two discrete backyard chicken farms. The three viruses formed a distinct cluster, with a similarity ranging from 99.1% to 100% among each other, but distinct from the previously identified lineages (16% difference from the closest lineage and 20% differences from the locally used vaccines). Therefore, these viruses were designated as a novel lineage in GI (GI-30).

GII-2: In 2017, a novel IBV strain (D181) was initially discovered in layer flocks experiencing a significant decline in egg production in the Netherlands [167]. Based on S1 gene sequencing, Strain D181 displayed a close relationship to the GII-1 strain D1466, exhibiting a 90% similarity. Consequently, D181 has been classified as a distinct lineage within the GII genotype, known as GII-2. Furthermore, D181 is considered a novel serotype as it differs antigenically from D1466, with only 9% crossneutralization observed between the two strains. Like D1466, D181 is primarily isolated from layer and breeder flocks, whereas occurrences in broiler flocks are rare. Infection with D181 is associated with high mortality rates and a decline in egg production without apparent respiratory signs [167]. Currently, D181 has become the second most prevalent IBV strain in the Netherlands and is frequently reported in Germany and Belgium [129].

GVII-1: In 2016, the novel IBV strain I0636/16 (GVII-1) was isolated in south China and recently identified as an emerged recombinant strain with a novel spike gene in a GI-28 (LGX/111119) backbone [168]. These findings emphasize the lack of protection of the currently used vaccines against GI-28 and suggest the circulation of a yet identified genotype under the detection limit of the surveillance. Although pathogenic to SPF chicks, strain I0636/16 exhibited reduced affinity to the respiratory tract compared to its putative parent strain LGX/111119. Interestingly, strain I0636/16 did not spread, suggesting its limited competitiveness [168].

GVIII-1 and GVIII-2: Domanska-Blicharz et al. [169] reported the identification of the IBV strain (gCoV/ck/Poland/G516/2018) isolated from a layer flock in Poland in 2018, that exhibited a decline in egg production. Through analysis of the partial S1 gene sequence (1,073 nt), this virus was found to be distinct from all known genotypes (GI–GVII), with a maximum identity of 81.4% to the PA/1220/98 variant, previously classified as a UV [18, 74]. The authors suggested that PA/1220/98 and gCoV/ck/Poland/G516/2018 be potential candidates for separate lineages in a new genotype (GVIII), namely GVIII-1 and GVIII-2, respectively [169]. Following the application of an in-house PCR to detect this variant, three more viruses related to the same lineage were discovered. Interestingly, the analysis of the partial S1 gene sequences (from 754 to 1,133 nt) revealed the presence of three related IBV strains previously isolated in Germany and the Netherlands between 2010 and 2015. Subsequently, Domanska-Blicharz et al. [169] and Petzoldt et al. [170] reported the full S1 gene sequence of 10 IBV strains, showing a 92.2%–100% identity among themselves and an 80% identity to the PA/1220/98 variant. Furthermore, the reported IBV strains shared 91.5%–95.2% identity with the partial S1 gene sequences of the previously recorded Polish strains in 2018, confirming the emergence of GVIII-II. It is noteworthy that the late identification of GVIII-II (also known as IB80) was due to a mismatch with the primers used at that time. By using an IB80-specific qRT-PCR, the authors were able to detect IB80-like strains in several countries in Europe and the Middle East from 2015 to 2021. Similar to GII, IBV strains belonging to GVIII were primarily detected in diseased layer and broiler breeder flocks experiencing a decline in egg production and increased mortality rates [170].

Novel Mexican genotypes and lineages (GI-31 and GIX): Recently, Mendoza-González et al. [123] described novel IBV strains isolated from various regions in Mexico. The full S1 sequence analysis of six Mexican strains collected between 2007 and 2019 revealed a 6% amino acid intergroup divergence and a 25% amino acid distance from the nearest genotype, GI-27. The authors suggested classifying these viruses as a new lineage within GI, tentatively designated as GI-30. However, it should be updated to GI-31, as GI-30 was proposed in 2020 [124, 145]. Furthermore, the authors identified two isolates, Mex-12 and Mex-3009, which exhibited a close relationship to the Mexican UV 98-0748, displaying a 3% amino acid divergence and a 45% amino acid distance from the nearest GIV genotype. Based on these findings, the authors proposed a new genotype designation, initially named GVIII. However, it also should be updated to GIX, as GVIII was previously proposed in 2018 [169]. In addition, Mex-56-7 (isolated in 2007) and Mex-14P (isolated in 2020) were clustering together with a low intralineage amino acid divergence of 10% and a high amino acid distance from the nearest GVII (50%). The authors described these Mexican isolates as a new genotype designated as GIX-1 (should be updated to GX). It is worth noting that this proposed classification should be confirmed with at least three reported viruses [74], while only two S1 gene sequences are currently available in the database. Nonetheless, the long detection period (2007–2020) of these two viruses suggests possible circulation in Mexico, and further surveillance with optimized primer sets may reveal additional sequences. The widespread geographic distribution and collection of these viruses over time and diverse locations support their persistence in Mexican chicken flocks, rather than being considered sporadic strains.

Novel Ivorian variant: Recently, Bali et al. [145] reported a unique IBV variant strain D2334/11/2/13/CI/2013 isolated in Ivory Coast in 2013 from H120 vaccinated flocks showing respiratory and digestive signs. The variant strain D2334/11/2/13/CI/2013 clustered separately from other genotypes/lineages with maximum inter- and intragenotype amino acid identities of 78.84%, and 78.6%, respectively. The investigated variant also did not show evidence for recombination events. The authors considered this UV to be the first member of a new lineage in GI (GI-3I) [145]. However, according to the genotype/lineage classification system suggested by Valastro et al. [74] this Ivorian strain should be classified as a UV until further identification of at least two more related strains from different outbreaks to prove the existence of this new lineage.

## 4. Immunity of IBV

The immune response against IBV is complex. Bird susceptibility is influenced by the Major Histocompatibility Complex (MHC) genotype. MHC is responsible for binding the antigen epitope and presenting it to T-lymphocytes by specialized antigen-presenting cells, determining the generation of humoral (when the epitope binds to MHC-II) or cell-mediated (when the epitope binds to MHC-I) responses [171]. The quality of the adaptive immune responses against IBV varies according to the virus strain, dose, host susceptibility, and age of vaccination or infection [172].

### 4.1. Passive Immunity

Maternally derived antibodies (MDAs) are passively transferred from the vaccinated breeders to their progeny to provide early protection against infection. Whilst IgG are predominant in the egg yolk, IgA and IgM are mainly present in the egg white because of the mucosal secretion in the oviduct. It was recorded that approximately 30% of the maternal IgG is transferred from breeders to their newly hatched chicks, however, the transfer of maternal IgA and IgM was estimated to be less than 1% [173]. IBV-specific MDAs have been detected in the serum, tears, and trachea of the newly hatched chicks [173–175]. It has been shown that chicks with elevated levels of IBV-specific MDAs exhibited an excellent level of protection (>95%) when challenged with IBV Mass strain at 1 day of age (doa) but not at 7 doa (<30%) [176]. These protection levels were correlated to the level of the local respiratory antibodies, not to the level of the serum antibodies [176]. Serum antibodies, however, are suggested to offer protection of the internal organs (kidneys and oviduct). Vaccination of layer breeders with heterologous live and inactivated IBV vaccines provided titers of 9–10 log2 maternally derived D388 VN antibodies in the hatched chicks, giving partial tracheal protection and high renal protection against challenge with D388 serotype (QX genotype) at 6 doa [27].

The role of MDA in interference with live IBV vaccines is debatable. It was shown that intraocular vaccination of MDA-positive chicks by IBV/Mass at 1 doa hastened the depletion of the serum antibodies and failed to produce an adequate immune response [5, 176]. This indicates that the MDAs interfere with the vaccination at 1 doa when using the same IBV serotype used for the breeder flock immunization. Interestingly, vaccination of MDA-negative chicks was marginally effective [176] indicating that the immature status of the immune system is another factor that could contribute to reducing the efficacy of the vaccination at 1 doa [177, 178]. Nevertheless, 1-day-old chicks are routinely vaccinated in commercial poultry farms. On the other hand, other studies reported no negative effect of the MDA on the vaccination at 1 doa [179–181]. This is probably because the IgY in the yolk sac continues to be transferred for at least 48 hr after hatching, so MDAs are not necessarily at their peak level at 1 doa [182], allowing partial exposure of the immune system to the vaccine virus. Indeed, since MDA are predominantly located systemically, mucosal vaccination via the ocular-nasal route may allow viral replication at the site of viral entry (the Harderian gland and upper respiratory tract) before being subsequently neutralized by the MDA during the viremia stage. This process potentially results in the induction of a protective local immune response [180].

### 4.2. Innate Immunity

The innate immune response is the first line of defense that nonspecifically targets invasive pathogens as they enter the host [183]. After IBV infection, hyperplasia of goblet cells and alveolar mucous glands occurs, leading to seromucous nasal discharge and catarrhal exudates in the trachea [184]. After the loss of cilia, epithelial degenerative changes and depletion of goblet cells and alveolar mucus glands occur, and other immunological components become activated [184].

IBV antigen is first recognized by two independent innate mechanisms including TLR and RIG-1. IFN-1 was found to reduce viral replication and delay the onset and severity of the disease in chickens due to its antiviral action [185]. Cytokines such as IFN-1, IFN-*γ*, IL-1*β*, IL-6, IL-8, IL-12, and macrophage inflammatory protein-1*β* are early activated by the innate pathways, in addition to other innate immune responses such as phagocytosis, complement, inflammation, cell death, and antigen presentation result in the innate protection of the adjacent cells [16, 186–188]. Later, CD8*αα* and Granzyme homolog A were shown to be increased, which was correlated with a subsequent decrease in the tracheal lesion scores and viral load [187]. In addition, it was shown that the macrophage count in the respiratory tract increased after IBV inoculation [189, 190] and coincided with a decrease in IBV viral loads and the production of IL-1*β* [191]. Remarkably, the innate immune response may differ according to the IBV strain pathogenicity, tropism, and population diversity [192–194].

These innate immune responses create an ideal microenvironment for T-cell activation and migration to the target organs, serving as bridging factors for connecting the local innate and adaptive immune systems. Interestingly, a greater upregulation of the innate immune responses-associated genes in the trachea is stimulated with a heterogenous population of IBV vaccines compared to a homogenous vaccine population [193]. This may explain the wider spectrum of protection provided by heterologous versus homologous vaccination.

### 4.3. Humoral Immunity

Humoral immune response to IBV is evaluated by determination of the level of IBV-specific antibody titers, either systemic (in serum) or local (in tears, tracheal wash, or oviduct), by ELISA, VN, or HI. ELISA is a group-specific test that could detect the antibody response within one week after infection or vaccination. Later, VN or HI could be used for detection of serotype-specific antibodies [195]. Unlike the innate immune response, the humoral antibody levels and avidity are shown to be favored by the homogenous IBV vaccine population [193].

#### 4.3.1. Systemic Humoral Immunity

The primary IgM immune response, which peaks at 8–10 days after infection, is a fast, short-lived response that is the first to be detected in chicken serum (at 5 days after infection). [196]. Therefore, detection of IgM in serum indicates recent infection by IBV. IgG becomes the predominant immunoglobulin around 14 days after vaccination or infection and its level is sustained for a longer time. The secondary IgM and IgG immune responses peak at the same time, but IgM declines faster [196]. It is worth noting that the levels of systemic humoral antibody do not correlate to protection against respiratory infection [84, 197]. However, it plays a crucial role in preventing the spread of the infection to the internal organs such as kidneys and oviduct and thus protects against nephrosis and a decline of egg production and egg quality [198–202]. On the other hand, mucosal antibodies are believed to be more relevant to the protection of the respiratory tract [203, 204].

#### 4.3.2. Mucosal (Local) Humoral Immunity

The local secretory immune response is a fast and considerably transient response characterized by secretion of local IgA [172]. When the IBV vaccine is administered by eye drop, IBV replicates in the Harderian gland, which is the main contributor to the local immune response, resulting in the production of high amount of lachrymal IgA [204–210]. This explains why vaccination by eye drop is suggested to be more relevant to the production of protective immunity than the drinking water method.

The pattern of the IgA levels in the tracheal and lachrymal secretions differs according to the IBV strain and dose. A very early increase of IgA level has been detected in tears at 3 days postvaccination (dpv) with Ark-DPI strain, indicating that this increase is a mucosal T-independent immune response [211]. Then the levels of IgA decrease to a nonsignificant level at 17 dpv. It was also reported that tracheal and lachrymal IgA were detected at 4 dpv with H120 alone or with H120 and CR88 simultaneously at 1 doa, and reached the peak at 7 and 14 dpv, respectively [212]. Smialek et al. [181] showed that the 4/91 strain alone or in combination with the Ma5 strain induced a longer and higher level of tracheal IgA that continued to rise till 21 dpv, compared to that of Ma5 alone, which declined 14 dpv. Upon secondary exposure (revaccination or challenge), the local IgA titers decline, indicating partial neutralization of the IgA, whereas IgG antibodies become the most dominant isotype in plasma and lachrymal fluid [211, 212]. Lachrymal and tracheal IgG immunoglobulins have been suggested to be produced locally in addition to be transduced from serum [213]. Significant levels of IgG antibodies were detected in lachrymal fluids and oviduct washes at 7- and 23-days postinfection (dpi) [214].

The role of local IgA in protecting against IBV infection is controversial. Many studies have shown that high levels of lachrymal IgA correlated to the refractivity to reinfection [203, 215, 216]. This presumably explains why revaccination with or secondary exposure to homologous IBV strains does not induce a secondary local IgA immune response. Polymeric IgA is suggested to play an important role not only in the inhibition of the viral infection by preventing the virus entry at the mucosal surfaces, but also in reducing the viral shedding by neutralizing the viral particles budding from the infected cells [217]. Conversely, Gelb et al. [174] found that some chickens with high levels of lachrymal IgA were susceptible to infection by IBV, while some other chickens with low levels of lachrymal IgA were protected, suggesting that local IgA immune response is not the main mechanism responsible for the protection of chickens against IBV infection. Moreover, in a study evaluating the transcriptional profiling in the trachea of chickens vaccinated with attenuated IBV-Mass vaccine, a significant upregulation of IgG expression was observed in the absence of IgA upregulation after the primary and secondary immunization, indicating that IgA might not be important in local immunity and the IgG dominated in the local immune response provide protection from virus entry by neutralizing viruses [186]. The absence of IgA upregulation after the primary immunization conflicts with other studies established that IgA antibodies are produced in high levels in the tracheal mucosa after primary immunization while significantly decreased after the secondary immunization [211, 212]. The roles of IgG and IgA in mucosal immunity remain to be established.

### 4.4. Cell-Mediated Immunity

Cell-mediated immunity (CMI) plays a significant role in the control of IBV [172]. S1, N, and M proteins have been reported to induce cell-mediated immune responses associated mainly with CTL [63, 77]. CTL activity is MHC class I-restricted, and lysis of IBV-labeled target cells is mediated by CD8+CD4− T cells not by CD4+CD8− T cells [62, 218]. Moreover, the CD8+ T cell response is established in the blood and spleen before the serum IgG humoral response to IBV [219], supporting that CTL response is correlated to the early decrease of viral titers and clinical disease [16, 218, 220]. Adoptive transfer of CD8+ T cells and *αβ* T cells reduced the viral loads 5 days postchallenge. However, adoptive transfers of CD4+ T cells and *γδ* T cells decreased the viral load by less than 11%, and did not alleviate the clinical illness after challenge [62]. Nevertheless, CD4+ T cells also contribute to the control of viral infection as they may produce antiviral cytokines, resulting in increased B cell activity and triggering the proliferation, maturation, and functional activity of CD8+ CTLs [221, 222].

The response of CTL, measured in the spleen, lungs, or kidneys of chickens infected with IBV was detectable at 3 dpi and reached the maximum by 10 dpi, correlated to a significant decrease of clinical signs and clearance of IBV from lungs and kidneys [16, 223]. Furthermore, the CTLs in the tracheal mucosa were found to be significantly increased by 3 or 4 dpi, peaked at 5 dpi, and then declined to baseline levels by 14 dpi, suggesting that these locally infiltrating CTLs are involved in the clearance of IBV from the trachea in the early phase of infection [224].

Chhabra et al. [212] studied the CMI of H120 alone (group I) or combined with CR88 (Group II) at 1 doa followed by revaccination of the two groups with CR88 at 14 doa. They found a significant increase in the infiltration of CD4+ and CD8+ in the trachea in the first 2 weeks after initial IBV vaccination, with the overall patterns of CD8+ cells subpopulations more dominant than those of CD4+ cells in the two vaccinated groups. Moreover, CD8+ cells were significantly higher in group II than those in group I at 21 and 28 doa, correlated with greater ciliary protection, lower viral RNA load in the trachea and kidneys, and reduced histopathological lesions against IBV-Q1 challenge.

In another study comparing the CMI response of Ma5 and 4/91, given separately or combined at 1 doa, a faster stimulation of CMI associated with an increase of CD8+ T cells in the Harderian gland was recorded 3 dpv using Ma5 alone, whereas delayed stimulation of CMI characterized by higher infiltration of CD8+ T cells in the Harderian gland was recorded 14 dpv using combined Ma5 and 4/91. On the other hand, vaccination with 4/91 alone induced a stronger stimulation of splenic CD4+ T cells and B cells at 3 and 7 dpv, respectively, without a significant increase in CD8+ T cells [181]. The authors suggested the Ma5 alone elicited CMI more efficiently than the 4/91 strain, which is suggested to generate a broadly crossreactive immune response and elevated respiratory IgA production.

The role of memory CTL response against IBV remains unclear. Guo et al. [186] found no local memory CTL response after the secondary exposure to the same IBV strain attributing this to the complete protection by the local IgG antibodies. It seems that the local memory CTL response occurs only when the virus succeeds in penetrating the local innate and humoral immunity allowing the host immune system to recognize it and thus activate the secondary CTL response.

## 5. Vaccination

**Figure 6:**
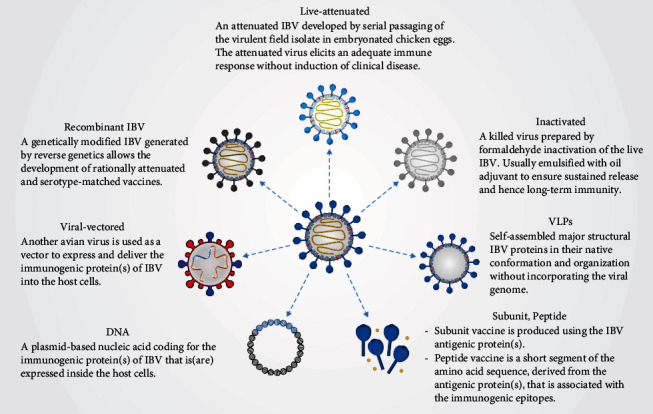
Types of infectious bronchitis virus (IBV) vaccines.

Vaccination is the major practical mean used for controlling IB. Live-attenuated and inactivated IBV vaccines are routinely used in commercial broiler, layer, and breeder chicken farms [172]. Next-generation IBV vaccines developed by novel methods have been raised in many studies but none of them is available commercially yet (Figure 6). This section provides an overview of the various types of IBV vaccines, explores the latest advancements in IBV vaccine research, and provides insights into the vaccination challenges against the emergence of new variants.

### 5.1. Live-Attenuated Vaccines

Live-attenuated vaccines have been extensively used to control IB worldwide. Live-attenuated vaccines are commonly used to immunize young broiler chicks and to “prime” future layers and breeders before administration of the inactivated vaccine [2, 172]. Live-attenuated IBV vaccines are developed by serial passaging the virulent field isolate in embryonated chicken eggs (ECE) [157, 225–228]. The mechanism of how the virulent IBV strain gets attenuated is unclear. However, it was suggested to be due to the mutations occurring during viral passaging particularly in the replicase gene [33, 157, 229, 230] and/or selection of an existing attenuated subpopulation in the original virus inoculum, known as “directional screening” [230]. Notably, the attenuation process in ECE leads to the generation of heterogeneous subpopulations within the vaccine batch that could also vary from batch to batch [111]. The live-attenuated virus should be able to elicit an adequate immune response without induction of respiratory illness in the vaccinated chickens. Preparation of a live-attenuated vaccine is expensive and time-consuming. A combination of heat treatment and egg passaging could achieve rapid attenuation of a field virus [231]. Live-attenuated IBV vaccines could be administered by mass application, via coarse spray and drinking water, or individually by eye drop, eliciting strong local and systemic responses of both humoral and CMI [2, 172].

In general, live-attenuated IBV vaccines provide excellent protection against homologous challenge [232, 233]. However, crossprotection against antigenically different strains is usually limited [234–236]. Thus, the choice of vaccine strain must be based on the strains prevalent in the country or the region. Higher levels of crossprotection against heterologous challenge could be achieved by a combination of two antigenically and serologically different vaccine strains such as “Ma5 and 4/91” [5, 27, 237] or “H120 and CR88” [238, 239]. This vaccination strategy is called “protecteoptype” [240]. The reason protectotype strategy gives a higher level of protection against heterologous challenge is not fully understood. However, it might be related to many factors including the vaccine titer, level and timing of vaccine replication in the chicken, and actual neutralizing titer [241]. Smialek et al. [181] suggested that the protectotype efficiency is due to the additive impact of Ma5 and 4/91 strains on different levels of both innate and acquired host immune response. Since the 793/B genotype (4/91 and CR88) is not licensed in many countries where no pathogenic field strains of the same genotype are circulating, some other vaccine combinations have been suggested [242–244]. However, they did not exhibit the same breadth of protection against multiple heterologous challenge. Another study showed that multiple vaccinations by the same serotype could reduce the viral load in chickens after heterologous challenge [245]. Crossprotection was shown to be limited by vaccination at 1 doa versus 10 or 14 doa, likely due to the lower level of antibody affinity maturation [246]. Simultaneous administration of three and even four different IBV vaccine types at 1 doa was shown to replicate to variable levels in the birds and provide adequate protection against homologous challenge with each type [247].

Nevertheless, many drawbacks have been reported about live-attenuated IBV vaccines. The tendency to revert back to virulence after back-passage in chickens holds a potential risk for these vaccine strains to induce outbreaks [117, 248–250]. Selection of a minor subpopulation of marginal virulence (less attenuation) within the attenuated vaccine was shown to occur in the chicken as early as 3 dpv leading to emergence of vaccine-derived strains of increased virulence [107, 112, 118, 251]. Moreover, genetic recombination between the vaccine and field strains of IBV promotes the creation of novel variant strains [98, 101, 117, 136, 156, 159, 164, 249, 250, 252–255]. The use of multiple live-attenuated vaccine strains could result in simultaneous recombination, even in the absence of field strains, leading to the creation of virulent recombinant viruses [165]. In addition, live-attenuated IBV vaccines could induce pathological lesions in the trachea and reduce the ciliary activity (ciliostasis), predisposing to secondary bacterial infection, especially in young chicks [256, 257]. Interference of the live-attenuated IBV vaccines with maternal immunity in young chicks has also been reported [5, 176]. Since the live-attenuated vaccines are developed by serial passaging in embryonated eggs, they become lethal to embryos and cannot be administered in ovo. In addition, research has demonstrated possible interference between live-attenuated IBV vaccines and other respiratory viral vaccines, specially Newcastle disease virus [258, 259].

### 5.2. Inactivated Vaccines

Inactivated IBV vaccines are routinely prepared by formaldehyde inactivation of the live IBV and emulsified with oil adjuvant to ensure sustained release and hence long-term immunity. Inactivated IBV vaccines are mostly used for immunization of layers and breeders, by injection, at least 4 weeks before the point of egg production [172]. Inactivated vaccines induce strong, long-lasting systemic humoral immunity required for the protection of the internal organs (kidneys and oviduct). Unlike the live-attenuated vaccine, the inactivated vaccine does not elicit strong cellular or local immune responses and thus, is not as effective in preventing the infection of the respiratory tract after homologous challenge [223]. Therefore, for an inactivated IBV vaccine to be effective, chickens must be “primed” with live-attenuated IBV vaccine at an interval of 4–6 weeks [260–263]. This ensures longer immunity and higher levels of circulating antibodies required for protection of the reproductive tract, as well as passage of maternal immunity from the hens to their progeny. Encapsulation of inactivated vaccine in chitosan nanoparticles allowed mucosal administration and elicited strong local IgA and IgG responses in addition to CMI in the trachea that provided effective protection against challenge [264]. Being unable to replicate, the inactivated vaccine does not regain pathogenicity or induce clinical disease in the vaccinated chickens [172]. Therefore, new IBV variant strains could be used for the preparation of autogenous inactivated vaccine without the risk of viral spread or induction of clinical disease in the vaccinated or nearby flocks. To increase the spectrum of protection, heterologous live priming followed by homologous inactivated boosting is suggested in many studies. Santos et al. [265] showed that the combination of heterologous live-attenuated vaccine (Massachusetts strain) with a booster dose of a homologous inactivated vaccine (BR-I IBV strain) two weeks later elicited mucosal and systemic memory immune responses, and provided protection against infection with the nephropathogenic homologous BR-I IBV strain. Similarly, heterologous priming with live vaccines MA5 and 4/91, followed by boosting with inactivated D1466 antigen provided protection against a drop in egg production and egg quality in the vaccinated layers challenged with virulent strain of D1466 [263]. Combination of inactivated antigens of different genotypes of IBV, or even two different diseases, into one vaccine without the risk of interference is another advantage of the inactivated vaccine [261]. Hassan et al. [266] demonstrated that priming with a bivalent live-attenuated vaccine of Mass and Conn types, followed by boosting with a bivalent inactivated vaccine of Mass and Ark types protected the hens from decline in egg production and reduced the viral replication and pathological changes in respiratory, renal, and reproductive organs after being challenged with the heterologous Canadian IBV DMV/1639 strain.

### 5.3. Viral Vectored Vaccines

Development of a viral vectored vaccine against IBV holds a great promise as an alternative to the live-attenuated IBV vaccine as it has the advantage of being safe, that is, overcome the problem of regaining pathogenicity and recombination with field viruses because it expresses only the immunogenic protein(s) of IBV without using the whole virulent organism. This also allows the vectored vaccine to escape the maternally derived immunity against IBV. However, the pre-existing or the maternally derived immunity against the viral vector itself could interfere with the viral vector replication and consequently hinder the expression of the IBV protein(s) leading to vaccination failure.

Some avian viruses including adenovirus [267–269], poxvirus [270–272], Herpes virus [273, 274], Newcastle disease virus (NDV) [275–279], and avian metapneumovirus [280] have been used as vaccine vectors for expressing IBV structural immunogenic protein(s) (S, S1, S2, or N protein). Among these virus vectors, NDV is of particular interest because of its respiratory tropism allowing it to elicit strong mucosal and cellular immunity at the site of viral entry [281]. Mass application of NDV-vectored vaccines makes them feasible for use in commercial poultry farms. In addition, NDV-vectored vaccines provide dual protection against IBV and NDV, another highly lethal avian virus of great concern, without the possible interference that may happen by coadministration of both IBV and NDV vaccines. Full S protein has been shown to be the antigen of choice for expression by NDV vector rather than expression of S1 or S2 alone [275] and modifications of the S protein could alter its protective efficacy [278]. The protective efficacy of NDV expressing full S protein of IBV is shown to be enhanced by vaccination at a higher age (4-week-old) [275] or by prime-boost vaccination at 1 and 14 doa [278] resulting in alleviated clinical signs and significant reduction of tracheal viral shedding. In another study, prime-boost vaccination (at 1 and 14 doa) or single vaccination at 10 doa using NDV expressing ectodomain of IBV S protein clearly showed reduced respiratory signs and induced better immune response versus single vaccination at 1 doa, however, it did not reduce the viral load in tears [279]. Recombinant NDV expressing a multiepitope of IBV S1 protein provided 90% protection against virulent IBV M41, significantly reduced viral load in the trachea, and reduced the pathogenicity to the trachea as shown by decreased ciliostasis score [282].

In addition to expressing the IBV immunogens, viral vectors can be engineered to express cytokines for augmented immune responses. For instance, coexpression of cytokine adjuvants such as IF-*γ* and IL-18 along with the IBV S1 protein by poxvirus has revealed enhanced cellular and humoral immune responses and provided better protection than the expression of IBV S1 protein alone [270–272].

Despite the promising results of the recombinant vectored vaccines, none of them has come to the market. The stability of the transgenes and the interference with vector-specific MDA, specially NDV, are of great concern. However, chimeric NDV vectors or increasing the antigenic dose were suggested to overcome the neutralization by MDA [283].

### 5.4. DNA Vaccines

A DNA vaccine is a plasmid-based or nonplasmid-based nucleic acid coding for the immunogenic protein(s) of IBV that is (are) expressed inside the hosts' cell. DNA vaccines induce enhanced cellular and humoral immunity especially when coadministered with DNA coding for cytokine adjuvants such as IL-2 [284] and granulocyte-macrophage colony stimulating factors [285]. A trivalent IBV DNA vaccine encoding for S, M, and N proteins has been shown to provide better protection than a monovalent vaccine expressing only one of these proteins [286–288]. Boosting with an inactivated vaccine enhances the protective efficacy of the DNA vaccines [286, 287]. Polyepitope-based DNA vaccine containing B and T cell epitopes derived from S1 genes of three IBV strains, namely, SH1208, Australian T, and Holte, protected against a lethal dose of the IBV SH1208 strain [289]. In a similar study, a chitosan-encapsulated DNA nanovaccine expressing IBV neutralizing epitopes (derived from S protein) and T-cell epitopes (derived from N protein) provided partial protection against challenge with IBV QX-like strain by reducing the viral load in kidneys and bursa of Fabricius, but not in the respiratory tract [290]. The authors attributed the incomplete protection to the parenteral administration of the vaccine which failed to elicit mucosal immunity at the respiratory tract. Moreover, the individual administration of DNA vaccines in general makes it difficult for application in large-scale poultry productions. However, in ovo vaccination at the hatchery is an alternative method that could be used for mass application of a DNA vaccine [291]. In addition, for mass application via drinking water, bacterial delivery systems were developed using attenuated *Salmonella* Typhimurium [292] and attenuated *Salmonella* Gallinarum [293] to deliver DNA vaccines carrying S1 and/or N genes and S gene of IBV, respectively. *Lactococcus lactis* was also used as a bacterial delivery system of the polypeptide-based IBV-DNA vaccine by drinking water or intranasal immunization [294, 295]. In addition, a nanoparticle adjuvant composed of Quil-A and chitosan (QAC) encapsulating DNA plasmid expressing the N protein of IBV Ark DPI strain (pQAC-N) offered a convenient intranasal application, elicited a robust cellular immunity and protected the chickens against homologous IBV challenge after prime-boost vaccination at 1 and 14 doa [296]. In a recent study, Chandrasekar et al. [297] showed that using the pQAC-N DNA vaccine as a prime dose before boosting with Modified Vaccinia Ankara (MVA) expressing the same N protein out-performed the prime-boost vaccination with MVA-vectored vaccine. This strategy elicited a robust local CMI in the lungs (lymphocyte proliferation) and led to a significant reduction of clinical severity and viral shedding in lachrymal fluid and tracheal swabs.

Since the DNA vaccine does not interfere with the MDA, it could be used in vaccinating young MDA-positive chicks. A DNA vaccine has other advantages including safety, stability, scalability as well as the ability to express multiple antigens. However, it needs to be administered in multiple doses or boosted with other kinds of vaccines such as inactivated and vectored vaccines.

### 5.5. Subunit, Polypeptide, and VLP Vaccines

A subunit vaccine is produced using the antigenic protein. A peptide vaccine is a short segment of the amino acid sequence, derived from the antigenic protein, that is associated with the immunogenic epitopes. The S1 and N proteins of IBV contain immunogenic epitopes responsible for the induction of neutralizing antibodies and CTL responses [1, 63, 64, 298]. It has been shown that the Spike ectodomain subunit vaccine provided significant protection against IBV challenge, whereas the S1 subunit vaccine provided inadequate protection, indicating the contribution of the S2 protein to bind to chicken tissue [299]. A polypeptide IBV vaccine based on multiple epitopes from S1 and N proteins was developed by expression in *E. coli*. Chickens immunized by the purified polypeptide vaccine showed significant humoral and cellular immune responses with 80% protection after challenge with the nephropathogenic IBV SAIBK strain [300]. Coadministration of S1 and N subunit vaccines derived from virulent IBV GX-YL5 strain was shown to provide better protection against homologous challenge than that of S1 or N proteins alone, but still lower than the protection afforded by the H120 inactivated vaccine [301].

VLPs are developed by the assembly of the major structural viral proteins in their native conformation and organization without incorporating the viral genome [302]. This technology makes use of the immunogenic properties of a live virus without the potential risk of regaining pathogenicity or recombination. The presentation of the immunogenic proteins in the native structure and morphology ensures exposure of the immunodominant epitopes and thus stimulation of neutralizing immune responses. It has been reported that IB VLPs could be formed using E proteins only [303], however, the efficiency was extremely low. IB VLPs, assembled by M and S proteins alone, effectively induced a humoral immune response similar to that of an inactivated vaccine, in addition to a significantly higher cellular immune response [304]. Moreover, VLP carrying the M, E, and S proteins of the IBV were generated using a Baculovirus system and were able to elicit IBV-specific antibodies and neutralizing immune response in chickens [305]. In another study, Lv et al. [306], constructed chimeric VLPs, composed of the M1 protein from avian influenza (AI) H5N1 virus and the IBV S1 protein fused to the transmembrane and cytoplasmic domains of AI H5N1 NA protein. Vaccination with these chimeric VLPs induced significantly higher S1-specific antibody response and levels of neutralization antibodies when compared to inactivated H120 virus in SPF chickens in addition to higher IL-4 secretion in mice. Other chimeric IB-ND VLPs carrying the IBV M protein and two recombinant proteins, IBV S1 and NDV F ectodomain, both separately linked to transmembrane and carboxyl terminal domains of IBV S protein, were constructed using a Baculovirus expression system. These chimeric IB-ND VLPs were shown to induce better humoral and cellular immunity responses compared to inactivated vaccines, and provided protection against both IBV and NDV [307].

Since the S protein of IBV is highly glycosylated and the neutralizing epitopes are highly conformation-dependent, the lack of glycosylation and the improper protein folding in the expression host could affect the immunogenicity of the expressed IBV protein and hence decrease the efficacy of the vaccine. In addition, the purification of the antigenic proteins or VLPs in large amounts and the individual administration by injection are two major limitations of the subunit, polypeptide and VLPs vaccines. These limitations make these types of vaccines uneconomical and inconvenient for application in large-scale poultry production.

### 5.6. Reverse Genetic Vaccines (Recombinant IBV)

Generation of recombinant IBV by reverse genetics allows understanding the function of the viral proteins and their manipulation for attenuation or changing the antigenic specificity. For example, deletion of 3ab and/or 5ab resulted in a mutant virus with an attenuated phenotype that could induce protection in chickens [40]. Exchange of the ectodomain of the S protein of the apathogenic Beaudette strain (Beau-R) with that of the pathogenic strains M41 [308] or 4/91 [309] induced protection against homologous challenge with M41 or 4/91, respectively, without increasing the virulence, while providing limited protection against heterologous challenge with the QX strain [236]. However, exchange of only the S1 or S2 subunits of the Beau-R with the corresponding regions from the pathogenic M41 provided significantly lower levels of protection compared to replacing the full ectodomain of the S protein [310]. On the other hand, substituting the S1 of IBV H120 with the virulent IBV strain SC021202 [311] or the nephropathogenic QX-like ck/CH/IBYZ/2011 strain [312] resulted in a nonvirulent recombinant IBV vaccine that could resist the challenge by SC021202 strain or QX-like strain, respectively. The limited replication of the Beau-R is suggested to be the reason of the incomplete protection when using the Beau-R as a vaccine backbone. In a recent study, Ting et al. [58] have shown that a prime oculonasal immunization of 1-day-old SPF chicks using live recombinant Beaudette strain expressing the S1 subunit of the QX-like strain KC boosted by intramuscular injection of an inactivated vaccine of the same virus at 14 doa provided better protection against challenge with virulent IBV of both QX and M41 strains. Weng et al. [313] replaced the spike gene of H120 with that of the QX strain, generating the recombinant virus H120-QX(S) that was apathogenic in SPF chickens and provided 100% protection against challenge with the virulent QX strain. All these studies emphasize that the S protein is not a determinant for IBV pathogenicity and could be used to precisely develop avirulent serotype-matched IBV vaccine candidates by reverse genetics.

On the other hand, switching the replicase gene of the virulent M41 with that of a Beaudette strain while keeping the remaining M41 genome unchanged resulted in avirulent chimeric IBV [32]. Later, Keep et al. [314] discovered a temperature-sensitive criterion of the recombinant IBV (Beau-R) determined by its replicase gene, emphasizing the role of the replicase gene in pathogenicity [229, 230]. The temperature-sensitive Beau-R was able to replicate at 37°C (the very upper respiratory tract) but not at 41°C (the core body temperature of chickens), presenting a new insight into the rational attenuation of IBV strains. Recently, Keep et al. [34] developed a rationally attenuated IBV vaccine candidate by substituting four amino acids in nsps 10, 14, 15, and 16, that was stable for up to 10 passages in primary CK cells and TOCs. In ovo application of the developed vaccine candidate showed successful hatchability rates (97% at a low dose; 10^1^ EID_50_, and 88% at a high dose; 10^4^ EID_50_), offering another advent for mass application in the hatchery.

Once an IBV reverse genetic system is set up, this technique allows the development of attenuated serotype-specific vaccine candidates against newly emerged variants more quickly than the attenuation of the pathogenic variant strain, through serial passaging in ECE. In addition, targeted RNA recombination based on the virulence genes allows the development of rationally attenuated IBV vaccine candidates rather than random attenuation by egg adaptation. Moreover, swapping only the S protein genes while keeping the remainder of the vaccine candidate constant, will help in the general predictability of the IBV vaccine candidates, rather than using different IBV vaccines that differ in many positions outside of the S gene [241]. Nevertheless, the chances of mutations and recombination are still possible with long-term use of recombinant IBV vaccines.

### 5.7. Vaccination Challenges against the Emergence of New Variants

Despite the implementation of various vaccination programs, IBV continues to circulate, evolve, and trigger outbreaks worldwide. The heterogeneity of IBV serotypes/genotypes poses a significant challenge in disease control by vaccination. In addition, the cocirculation of more than one serotype/genotype in the same geographical area facilitates the continual emergence of new variants by genetic recombination. When facing a newly emerging variant, the decision must be made whether to develop a homologous vaccine, rely on the currently available strains within the geographical area or introduce a new vaccine strain. Developing a homologous live-attenuated vaccine for each emerging variant is impractical as it is expensive and time consuming. In addition, differentiating between vaccine and field strains (DIVA) in diagnostics becomes complicated [111]. Moreover, using a homologous vaccine adds complexity to assessing viral evolution as reisolation of the vaccine strain will yield an apparently low evolution rate [117]. On the other hand, relying on the “protectotype” concept (using two antigenically distinct vaccine strains to provide broad protection against heterologous challenge) may allow DIVA but might not be as efficient as the homologous vaccine [235]. In addition, possible recombination between multiple vaccine strains could further complicate matters. Interestingly, a molecular epidemiology study conducted in integrated poultry companies in northern Italy revealed that the replacement of heterologous protectotype vaccination, based on Mass and 793B strains, with a homologous QX vaccine (D388) led to a higher vaccination pressure [113]. This phenomenon occurs because the immunity elicited against IBV is not sterilizing, allowing the field strains to replicate and evolve in partially protected chickens. The highest pressure was primarily directed toward the RBM in the S1 CTD, which is a major target for neutralizing antibodies. This homologous vaccine pressure potentially influences the emergence of vaccine-immunity escape mutants. By contrast, the heterologous vaccination is suggested to induce a more diversified spectrum of immune responses, making it more challenging for specific immunity escape mutants to be selected [113]. Although the protectotype concept based on Mass and 793B types demonstrated a broad spectrum of protection against multiple variants, the decision to introduce the 793B type vaccine into a region where this serotype has not been reported before necessitates thoughtful consideration. Once a new strain is incorporated into the vaccination strategy, withdrawing it becomes challenging. With long-term use, vaccine-derived strains with virulent characteristics may evolve and trigger outbreaks [136–138]. Therefore, there is an urgent need for a novel vaccine platform other than live attenuated vaccines to halt the continuous emergence of variants [315].

In field application, the efficacy of a vaccine may be influenced by various factors, leading to outcomes that may differ from the results observed in controlled laboratory settings. Several practical considerations, such as the poultry management practices, bird health status, vaccination timing, handling, storage, route of administration, and the presence of diverse circulating viral variants, can all play a crucial role in shaping the actual effectiveness of the vaccine in the field. Even with the best-matched vaccine in use, suboptimal vaccination practices can result in inadequate immunization, ultimately leading to vaccination failure. For instance, standardizing the hatchery application of the vaccine is essential to ensure comprehensive coverage of the chicks. Proper adjustment of the spray equipment, adhering to the recommended droplet size, prevents vaccine loss to the environment or excessive inhalation into the lower respiratory system. Gel administration method has been proposed to be equally effective in hatchery vaccination against IBV, without the spray-associated issues [316]. In addition, maintaining suitable water quality and temperature is crucial, as these factors can significantly impact vaccination effectiveness. Another aspect to consider is the potential interference from the simultaneous application of live vaccines for other diseases [259]. Taking all these factors into account is vital to achieve the desired outcome of vaccination in the field.

## 6. Conclusions

IB is an extremely contagious viral disease that negatively impacts the poultry industry around the globe. While IBV replicates primarily in the respiratory epithelial cells, certain viruses exhibit broader tropisms, disseminating to internal organs specially the kidneys, oviducts, and proventriculus. Therefore, local respiratory immunity is vital in preventing viral infection, while systemic immunity protects the internal organs and ensures transmission of maternal antibodies to the newly hatched chicks. The global landscape of IBV is characterized by the presence of multiple serotypes/genotypes, regional distribution patterns, and the continuous emergence of novel variants. The use of live vaccines can significantly impact the geographical dynamics and distribution of IBV genotypes.

The diversity of IBV arises from its high mutation rate and recombination, creating variants with distinct characteristics in tropism, virulence, and/or vaccine coverage. Current IBV vaccines, while offering clinical protection, allow the virus to replicate under immunization pressure, leading to evolutionary changes, and escape from vaccine immunity. In addition, these vaccines can recombine with wild-type viruses, giving rise to novel variants. This is how IBV wins the battle against the current vaccines making the long-term use of such vaccines unsafe. Alternative vaccine platforms such as DNA, peptide, subunit, VLP, and viral-vectored vaccines were proposed and tested in many experimental studies. Although they offer a safety advantage over the live-attenuated vaccines, they also come with their own limitations.

## 7. Future Perspectives

Control of IBV is extremely challenging due to its highly evolving nature and diverse antigenicity. Addressing these challenges requires careful consideration of vaccination strategies. Vaccines that prevent infection entirely offer a perfect solution, capable of eradicating the disease. However, current IBV vaccines, providing partial protection by reducing viral replication without preventing infection, can sometimes exacerbate the situation. Although some vaccines may appear satisfactory at the clinical level, effectively protecting flocks from clinical disease, their protection is transient. With the emergence of a novel variant, severe outbreaks can occur, as these vaccines do not stop the virus replication and shedding. Thus, the evaluation of the protective efficacy of IBV vaccines should include both clinical and viral shedding parameters.

Research efforts should be directed toward the development of novel vaccines that effectively and safely prevent infection. The future IBV vaccine needs to meet several criteria to efficiently control the disease. An ideal IBV vaccine should provide broad-spectrum protection, elicit both local and systemic humoral and cellular immune responses, circumvent the maternal antibodies, and be cost-effective, convenient for mass application in the field, and safe. Research in this area should be coupled with deep exploration of viral evolution, immunogenicity, pathogenicity, host genetics, and innate immunity. This interdisciplinary approach can lead to new avenues for development of effective vaccines.

Continuous surveillance with up-to-date diagnostic tools is crucial to understand the epidemiology worldwide and promptly detect emerging genotypes/lineages. While S1 gene sequencing is currently sufficient for IBV genotype identification, there is a growing need for full genome sequencing to comprehensively explore the evolutionary trajectories of IBV variants.

Implementing stringent biosecurity measures and sound management practices are essential to prevent the introduction and spread of infection. In addition, strict adherence to the proper vaccination procedures is vital to ensure the maximum vaccine coverage and an optimum immune response. Therefore, vaccination should be complemented with a holistic strategy that combines robust surveillance systems and biosecurity measures, recognizing that it does not stand alone in the battle against IBV.
